# Assessing the relative importance of health and conformation traits in the cavalier king Charles spaniel

**DOI:** 10.1186/s40575-017-0056-2

**Published:** 2018-01-23

**Authors:** Katrien Wijnrocx, Liesbeth François, Peter Goos, Nadine Buys, Steven Janssens

**Affiliations:** 1 KU Leuven Department of Biosystems, MeBioS, Kasteelpark Arenberg 30, box 2456, 3001 Leuven, Belgium; 2 Department of Engineering Management, University of Antwerp, Prinsstraat 13, 2000 Antwerp, Belgium; 3 KU Leuven Department of Biosystems, Livestock Genetics, Kasteelpark Arenberg 30, box 2456, 3001 Leuven, Belgium

**Keywords:** Choice experiment, Selection, Weighted index, Dog, Syringomyelia, Mitral valve disease

## Abstract

**Background:**

The selection of a future breeding dog is a complicated task, in which disease characteristics and different traits have to be combined and weighed against one another. Truncation selection, that is the exclusion of affected animals, may be very inefficient when selecting on a large number of traits, and may result in a reduction of the genetic diversity in a population or breed. Selection could be facilitated by the use of a selection index that combines multiple traits or breeding values into one score. This however requires a consideration of their relative value according to their economic weight, which is difficult to express in monetary units for health traits. The use of a choice experiment to derive non-market values might be a solution to this problem. This is a pilot study to assess the potential use of choice experiments to ascertain the public preference and relative importance attached to health- and conformation traits in the selection of a Cavalier King Charles spaniel. The focus was on two prevalent disorders, mitral valve disease and syringomyelia, and on several important conformation traits such as muzzle length and eye shape. Based on available prior information, a Bayesian D-optimal design approach was used to develop a choice experiment and the resulting choice sets.

**Results:**

Every participant (breeder or owner) in the choice experiment was presented with a total of 17 choice sets, in which at most four traits could vary to reduce the cognitive burden. A total of 114 respondents participated in the choice experiment and results showed that respondents (breeders/owners) current attitudes were directed towards health (syringomyelia and mitral valve disease), followed by eye shape and level of inbreeding.

**Conclusions:**

This approach identifies the value breeders and owners attach to certain traits in the breeding objective. The resulting relative weights, represented as the logworths obtained from the choice experiment, could be an alternative to economic weights. They could be implemented as a weight when breeding values are available, but more study on this topic will be necessary. A challenge in this approach is to scale up the experiment with additional traits. Moreover, for other traits, the genetic parameters and correlations should be known first, in order to include them in the health selection index as well.

## Plain english summary

When selecting a future breeding dog, different disease characteristics and other traits have to be balanced against one another, which makes it a complicated task. In the case of selecting for a large number of traits, the exclusion of all affected animals might be very inefficient, since this may reduce the genetic diversity in a population or breed. A solution could be the use of a selection index, in which all traits of interest are combined into one single value according to their relative weight. For this however, traits need to be evaluated according to their economic weight, which is difficult for health traits. This study examined breeders and owners attitudes and the importance they attached to three health- and three conformation traits in the selection of a Cavalier King Charles spaniel, and this using a choice experiment. The focus lay on two very prevalent disorders, mitral valve disease and syringomyelia, and on several important conformation traits such as muzzle length and eye shape. Results indicated that breeders and owners valued health traits highest followed by eye shape and level of inbreeding.

## Background

Many modern dog breeds suffer from multiple heritable diseases with varying prevalence and severity. The choice of potential breeding animals involves the consideration of a multitude of traits and disease characteristics. The combination of all desired characteristics seriously complicates the selection of potential breeding animals. Selection is further complicated by the need to maintain the genetic diversity of a breed as the exclusion of animals with any undesired trait will lead to a genetic bottleneck in the breeding population [1].

The Cavalier King Charles spaniel (CKCS) breeders have been strongly criticized since the BBC documentary “pedigree dogs exposed” (BBC, 2008). CKCS are prone to several genetic diseases, such as mitral valve disease (MVD) and syringomyelia (SM). MVD is a heart disease in which the mitral valve closes insufficiently leading to mitral regurgitation [2, 3]. A higher prevalence and earlier onset of MVD have been reported frequently in CKCS compared to other dog breeds [2, 4, 5]. SM on the other hand is a neurological condition that frequently occurs in CKCS and is characterized by the formation of fluid-filled cavities within the spinal cord [6]. Other traits of interest within the CKCS breed are the shape of the eyes, the length of the muzzle, and the colour of the dog. Also the level of inbreeding could be important, due to the more general concern about the levels of genetic diversity in many dog breeds [7, 8].

The use of a selection index would aid the search for the most optimal breeding animal as it combines health and conformation traits into one score by considering the relative values of each trait according to their economic weight [9]. However, it is not always possible to quantify the relative value of certain traits, such as animal health and welfare traits, in monetary units. The addition of non-market values to such traits in the breeding program could alleviate this problem with the total genetic gain of a trait as the sum of both the non-market and the market genetic gain [10, 11]. A choice experiment is one method to derive non-market values. Choice experiments are frequently used in the fields of marketing and economics and aim to quantify the preferences of an individual for certain attributes or goods [12]. In dog breeding this technique has never been employed before and it might help determine the breeders’ and owners’ preferences for breeding goal traits. In livestock breeding, this technique has already been used to derive breeding goals, for example in sheep breeding [13]. Generally, respondents in choice experiments are asked to choose between certain alternatives of a good (in this case a dog) having different attributes (in this case different traits). The alternatives, also termed profiles, are presented simultaneously to a respondent as a choice set. Every alternative is presented as a combination of different levels of the attributes [13, 14]. To reduce the complexity for respondents, it is common to present only a subset of the traits within any given choice set. This kind of choice experiment is called a partial profile choice experiment [15, 16].

This paper assesses the potential usefulness of a choice experiment to ascertain the relative importance breeders and owners attach to different traits within the Cavalier King Charles spaniel (CKCS) This study originated as a response to a recently invoked law that will oblige Flemish CKCS breeders to screen their dogs prior to breeding. We believe this pilot study is the first to investigate the potential of choice experiments in the domain of dog breeding.

## Methods

Conducting a choice experiment consists of different steps. First, the attributes (traits) and attribute levels need to be defined. Secondly, a suitable experimental design needs to be constructed: alternatives need to be selected for presentation to the respondents and they need to be combined in choice sets in order to generate as much information as possible concerning the respondents’ preferences. Next, the questionnaire has to be developed. Finally, after collecting the responses, they need to be analysed statistically using a suitable choice model [12].

### Identification of attributes and attribute levels

The potential traits of interest to CKCS breeders, including general characteristics of the breed standard as well as disease traits, were listed in consultation with stakeholders and experts in CKCS breeding, including the Belgian Cavalier Kennel club and the president of the Cavaliers for Life foundation. As the number of attributes in a choice experiment is limited to reduce the cognitive burden of the respondents [15], only following attributes were considered to be of major interest in the choice of a CKCS: shape of the eyes, coat colour, muzzle length, level of inbreeding, purchase price of the dog, syringomyelia status, eye disease status, and mitral valve disease status. The different attribute levels (trait levels) are listed in Table 1. All attributes have three levels, except for coat colour (2 levels), level of inbreeding (4 levels), and price (4 levels).

**Table 1:** Different attributes (traits) and levels considered in the choice experiment

Attribute	Level
Coat colour variety^a^	1. Within the same colour line
	2. Between colour lines
Eye disease	1. Not tested
	2. Tested and clinical symptoms present
	3. Tested and free
Eye shape	1. Walleyed
	2. Small
	3. Prominent
Level of inbreeding	1. 0–3%
	2. 3–6%
	3. 6–9%
	4. 9–12%
Mitral valve disease	1. Not tested
	2. Tested and clinical symptoms present
	3. Tested and free
Muzzle length	1. 34–38 mm
	2. 38–42 mm
	3. 42–46 mm
Price	1. €700 - €900
	2. €900 - €1100
	3. €1100 - €1300
	4. €1300 - €1500
Syringomyelia	1. Not tested
	2. Tested and clinical symptoms present
	3. Tested and free

^a^Within the same colour line means wholecolor x wholecolor or particolor x particolor breedings, between color lines means wholecolors x particolors breeding. Wholecolor = Black& Tan or Ruby, particolor = Blenheim or Tricolor

### Experimental design

For the design of the choice experiment, the module “Design of experiments” of the JMP software was used (JMP version 12, SAS Institute Inc., Cary, NC, 1989–2007). Based on the multinomial logit model for choice data, a Bayesian D-efficient partial profile design was constructed, optimised for 17 choice sets. This D-efficient design takes into account the available prior knowledge about the preferences of the respondents. Thereby, the D-efficient design avoids uninformative choice sets in which one alternative completely dominates the other [15]. A pilot study provided the prior information required to generate the final Bayesian D-efficient choice design. The final choice experiment presented the respondents with 17 choice sets of two alternatives. The alternatives within each choice set were a combination of the 8 attributes listed in Table 1. They were presented as partial profiles meaning that at most 3 or 4 attributes varied within each choice set, thereby limiting the cognitive burden for the respondents [15].

### Questionnaire development

The choice experiment was presented as a questionnaire. We provided respondents with a web link, allowing them to carry out the experiment at their convenience. Questionnaires were sent via email by the Belgian Cavalier club “Pejatoyspa” to a sample of breeders and owners, and it was advertised on the website of the Cavaliers for Life foundation. To help respondents, we presented an explanation of the experiment to familiarize them with a choice experiment and the 8 selected attributes and their levels. We asked the respondents to choose between two different dogs they would use as a breeding animal, displaying a number of attributes. Additionally, general information was collected about the respondents’ age, sex, country of origin, occupation (owner, breeder, or show judge), the number of dogs owned, the number of CKCS owned, and whether their CKCS is affected by one of the diseases. To avoid fraudulent respondents, a captcha verification was used before starting the questionnaire.

### Statistical analysis

The data analysis was performed using the “choice modelling” platform in JMP (JMP version 12, SAS Institute Inc., Cary, NC, 1989–2007). The relative importance of each trait was quantified by estimating the multinomial logit model in the eight attributes using a maximum likelihood approach (the JMP software uses the Firth bias correction in its maximum likelihood procedure; see [17]).This results in an estimate of the utility the respondents associate with each attribute level, i.e., with each level of each trait [12]. The overall significance of each trait was evaluated using likelihood ratio tests. In the initial model, all attributes were included and tested, and only the significant attributes were retained in the final model. The relative importance of each trait was measured by the logworth statistic, this is the –log_10_ (*P*-value of the likelihood test) [18]. To test the possible effect of being a breeder, the interaction effect of the variable breeder was tested with all attributes.

## Results

### Response rate

Between March 2016 and September 2016, internet questionnaires from different CKCS owners and breeders were collected. The open distribution of the questionnaire did not allow a classical calculation of the response rate. However, a total of 207 respondents visited the website online, of which 114 fully completed the survey and 87 filled it in partially. Respondents had to make 17 choices each, which resulted in a dataset of 1938 choices. Only the fully completed questionnaires were considered for further analysis.

### Respondent information

One part of the survey was intended to collect generation information concerning the respondents. Out of the 114 respondents who completed the questionnaire, 103 (90.37%) were women and 10 were men (8.77%). One respondent did not fill in this question. Almost all respondents were dog owners themselves (111 respondents or 97.37%), 46 (40.35%) were also dog breeders, and 11 (9.65%) were show jury members. 86.85% (99) of the respondents had a CKCS at home, and 63% of these already had been confronted by a genetic disorder (heart disease, syringomyelia, eye disease, or a combination of these). Respondents originated from different countries, with 28 (25%) coming from Belgium, 20 (18%) from Denmark, 18 (16%) from USA, 22 (19%) from UK, 5 (4%) from the Netherlands and 19 (17%) from other countries. Two respondents (2%) did not fill out this question.

### Modelling results

At first, the effect of being an breeder was investigated by including interaction term of breeder × attribute in the model. None of these interaction terms reached significance (a value of 5% was utilized to determine the statistical significance, see Table 2). Further analysis therefore considered both groups together. The results of the initial and final multinomial logit model are presented in Tables 3 and 4, respectively. The initial model included all possible attributes, whereas the final model only included the statistically significant attributes (a value of 5% was utilized to determine the statistical significance). Both tables show the estimated marginal utility values (parameter estimates) for all different attribute levels, as well as their significance using a likelihood ratio test. The initial model shows that all attributes matter, except for the price of the dog. Therefore, the price attribute was excluded from the final model. The attributes are also ranked in order of importance. Note that the order of the importance changes between the initial and the final model, with mitral valve disease (MVD) being more important and inbreeding being less important in the final model than in the initial model.

**Table 2:** Multinomial logit model results for each trait, including the interaction with breeder

	L-R Chi^2^	DF	*P*-value
Eye shape	41.315	2	<0.001
Coat colour	5.963	1	0.015
Snout length	9.274	2	0.010
SM	182.589	2	<0.001
Eye disease	30.961	2	<0.001
MVD	34.310	2	<0.001
Inbreeding	32.243	3	<0.001
Price	2.478	3	0.479
Eye shape × Breeder	1.520	2	0.468
Coat colour × Breeder	0.477	1	0.490
Muzzle length × Breeder	0.163	2	0.922
SM × Breeder	0.523	2	0.770
Eye disease × Breeder	0.481	2	0.786
MVD × Breeder	0.639	2	0.726
Inbreeding × Breeder	0.847	3	0.838
Price × Breeder	0.323	3	0.956

*L-R Chi*^*2*^ likelihood ratio test, *df* degrees of freedom

**Table 3:** Initial multinomial logit model results for each trait. Traits sorted in decreasing order of importance

	Marginal utility values	L-R Chi^2^	DF	*P*-value	–log_10_ (*P*-value)
SM					
Tested & present	−0.605				
Not tested	−0.754	188.564	2	<0.0001	94.282
Tested & free	1.359				
Inbreeding					
0–3%	0.451				
3–6%	0.501	50.201	3	<0.0001	23.349
6–9%	−0.597				
9–12%	−0.355				
Eye shape					
Walleyed	−1.069				
Prominent	0.758	44.959	2	<0.0001	22.480
Small	0.491				
MVD					
Tested & present	−0.841				
Not tested	−0.681	38.605	2	<0.0001	19.302
Tested & free	1.522				
Eye disease					
Tested & present	−0.621				
Not tested	−0.258	32.793	2	<0.0001	16.396
Tested & free	0.879				
Muzzle length					
34-38 mm	0.536				
38-42 mm	0.041	10.368	2	0.006	5.184
42-46 mm	−0.577				
Coat colour					
Within lines	−0.369	6.014	1	0.014	4.255
Between lines	0.369				
Price					
€700 - €900	0.317				
€900 - €1100	−0.347	2.551	3	0.466	0.763
€1100 - €1300	−0.034				
€1300 - €1500	0.064				

*L-R Chi*^*2*^ likelihood ratio test, *df* degrees of freedom, *−log*_*10*_ *(P-value)* logworth statistic

**Table 4:** Final multinomial logit model results for each trait. Traits sorted in decreasing order of importance

	Marginal utility values	L-R Chi^2^	DF	*P*-value	–log_10_ (*P*-value)
SM					
Tested & present	−0.786				
Not tested	−0.530	261.153	2	<0.0001	130.577
Tested & free	1.316				
MVD					
Tested & present	−0.667				
Not tested	−0.646	108.581	2	<0.0001	54.291
Tested & free	1.313				
Eye shape					
Walleyed	−1.026				
Prominent	0.695	53.709	2	<0.0001	26.855
Small	0.331				
Inbreeding					
0–3%	0.318				
3–6%	0.556	53.378	3	<0.0001	24.908
6–9%	−0.447				
9–12%	−0.247				
Eye disease					
Tested & present	−0.534				
Not tested	−0.039	47.491	2	<0.0001	23.745
Tested & free	0.573				
Muzzle length					
34-38 mm	0.124				
38-42 mm	0.244	10.014	2	0.007	5.007
42-46 mm	−0.368				
Coat colour					
Within lines	−0.220	6.677	1	0.010	4.629
Between lines	0.220				

*L-R Chi*^*2*^ likelihood ratio test, *df* degrees of freedom, *−log*_*10*_ *(P-value)* logworth statistic

Comparing the logworths of the likelihood ratio test statistics provides a rough indication of the relative importance of each trait. When looking at the final model, it can be observed that syringomyelia (SM) and mitral valve disease (MVD) are the most important attributes in the choice of a dog. SM is even twice as important as MVD (logworth of 130 compared to 54 respectively). Three other traits, eye shape, level of inbreeding and eye disease, have an equal importance (logworths were all around 25), followed by muzzle length, and coat colour which had almost no importance (logworth of 5 only).

The marginal utility values for each trait show that, for the included disease traits, the values for “tested dogs free from the disease” are positive, whereas those for “tested and disease present” or “not tested” are negative. This indicates that participants attribute a higher utility value to a tested dog compared to both the non-tested animals and the tested animals that were affected by the disease. The marginal utilities for non-tested animals are less negative than for animals known to have a disease, indicating that the respondents prefer non-tested animals over animals with the disease. When comparing the values for the “tested and disease present” status among the included diseases, the marginal utility value for SM is highest, followed by MVD and eye disease, indicating that respondents value SM as the worst disease. Concerning the level of inbreeding, the marginal utilities become negative when exceeding an inbreeding level of 6% or more.

## Discussion

This study invited breeders and owners to express their attitudes towards the importance of traits in the selection of a CKCS by means of a choice experiment. This approach has its advantages. It presents the respondents with different alternatives and choosing the preferred alternative is a more realistic task compared to for example conjoint analysis, in which respondents are asked to rank the different profiles [13]. A critical aspect in the development of the questionnaire was the identification of the traits or attributes. It was important that respondents were presented with realistic alternatives to choose between. In this respect, consultation with stakeholders such as the Belgian Cavalier Kennel club and the Cavaliers for Life foundation, as well as the pre-testing in a small scale trial was very useful in specifying relevant attributes.

The final study was completed by 114 respondents, of which 90% were female. This bias is frequently seen in other questionnaires applied to dog owners [19–21] and can be explained by the predominance of women in veterinary and animal welfare-related fields [21]. Respondents were Belgian, Danish, UK and USA breeders and owners, which indicates an international evaluation of the relative importance of traits.

The choice experiment showed that no difference in preference between breeders or owners, they both attached most importance to the health traits such as SM and MVD. This was partly an expected result, as the CKCS breed is known to suffer from these diseases [2, 6]. Both SM and MVD are assumed to be complex diseases, in which the disease phenotype results from the interaction of multiple genes and the environment. Currently, many breeding organizations apply strict breeding rules against complex diseases and in some cases this has led to a reduction in prevalence of disorders and improved overall health status of the breed [22, 23]. However, choosing a breeding dog involves considering multiple traits and disease characteristics, because breeds are susceptible to a number of diseases [24, 25]. Therefore, it is necessary to assess the relative importance of each disease trait or breed characteristic, as well as the genetic correlations. This pilot study presents a tool to determine the relative importance of traits and can help to prioritize traits in a breeding program for the CKCS.

The importance that is attached to the health traits in this study is not surprising, given the importance that is attached to it in the breeding recommendations prior to breeding by the British Kennel Club (an assessment by the British Veterinary Association / Kennel Club breeding scheme). However, these preventive screening measures for SM and MVD are not compulsary. In Belgium, preventive measures are only recently mandatory and this only for half of the country (recommended by the Flemish government since 2016). Furthermore, the importance attached to health traits can also be reflected by the high prices of the treatment of these traits. For example SM can be treated medically or by surgical management, which is costly [26]. Our study also indicated that the “level of inbreeding” is considered important by breeders and owners. This is not surprising given the number of studies addressing the problems concerning low genetic diversity and inbreeding that pedigreed dogs suffer from. Several studies on the CKCS showed that the levels of inbreeding are low and effective population sizes are high compared to other breeds (effective population size of around 105) which indicates that inbreeding is not a considerable problem in the breed [27–29]. It was also demonstrated that the price of the dog does not seem to be of importance for respondents.

The choice experiment which was applied in this study was a first investigation in the rational thoughts of breeders and owners towards some non-economic traits such as desired conformation or beauty traits in the selection of a CKCS. A next step could be to include other possible traits of interest. Our study approach resulted in logworths, that give an indication of the relative importance of each trait and can be a first step towards creating a comprehensive total selection index, similar to a multi-trait index in cattle, which can be used to ameliorate the breed [30]. However, when creating an index, some additional challenges need to be resolved. The relative importance of each trait can be used as a direct value only when EBVs are available for this trait. This is currently not possible because some traits are not routinely recorded in the CKCS population, such as for example muzzle length or eye shape. For these traits, genetic parameters and genetic correlations should be estimated in order to develop a true selection index [9]. For the traits for which EBVs are available, such as MVD and SM, the estimated relative importance values in this study can be directly used to combine them into a selection index. However, to evaluate genetic progress, the genetic correlation has to be estimated.

There is an additional challenge when an overall index would become available. Ideally selection should be balanced with the rate of inbreeding, applying for example optimal genetic contribution. A step towards balanced breeding might be to restrict the use of sires at an international level. Regulations should be made to promote using seldom-used lines or animals, and to limit the use of popular sires. The use of popular sires has been shown to lead to a dissemination of genetic disorders [8]. Also monitoring of genetic diversity, with restriction of the rate of inbreeding is an important factor as demonstrated by different studies [31, 32].

## Conclusions

As a conclusion, this study assessed the priorities and relative importance of traits in the selection of a CKCS as breeding or companion animal using a choice experiment. The marginal utility of each trait can be used as a weighing factor to define a total selection index, including health- and non-economic traits.

## Abbreviations

CKCS: Cavalier King Charles spaniel
CM: Chiari-like malformation
EBV: Estimated breeding value
MVD: Mitral valve disease
SM: Syringomyelia
